# Incomplete Presentations in Typical Chronic Inflammatory Demyelinating Polyneuropathy: A Single‐Center, Retrospective Study

**DOI:** 10.1002/mus.28419

**Published:** 2025-04-22

**Authors:** Young Gi Min, Irad Ahmed, Christina Englezou, Yusuf A. Rajabally

**Affiliations:** ^1^ Department of Translational Medicine Seoul National University College of Medicine Seoul Republic of Korea; ^2^ Inflammatory Neuropathy Clinic, Department of Neurology University Hospitals Birmingham Birmingham UK; ^3^ Aston Medical School Aston University Birmingham UK

**Keywords:** chronic inflammatory demyelinating polyneuropathy, course, incomplete, phenotype, response, treatment, typical

## Abstract

**Introduction/Aims:**

Incomplete forms of typical chronic inflammatory demyelinating polyneuropathy (CIDP) have recently been described, but their frequency and clinical characteristics are uncertain. This study aimed to describe a cohort of patients with incomplete typical CIDP.

**Methods:**

We retrospectively analyzed 64 consecutive treatment‐naïve patients with CIDP. Phenotypes were classified based on detailed motor examinations, and clinical, electrophysiological, and therapeutic characteristics were compared.

**Results:**

Nineteen (30%) subjects with typical CIDP presented with an incomplete phenotype; 12 (63.2%) exhibited a proximal arm‐sparing pattern, 3 (15.8%) a distal arm‐sparing pattern, 3 (15.8%) a pure paraparetic form, and 1 (5.2%) had a pure proximal form. In cases without full motor recovery, 11 (68.8%) maintained their original phenotype, while the rest transitioned to the complete (18.8%) or to another incomplete form (12.5%) due to involvement of previously unaffected segments. Subjects with incomplete typical CIDP had milder pre‐treatment disability and weakness compared to those with the complete form, while other clinical and electrodiagnostic features were comparable. As opposed to the complete form, disability in incomplete typical CIDP at diagnosis showed no correlation with muscle strength.

**Discussion:**

Incomplete forms were observed in nearly one‐third of subjects with typical CIDP. Incomplete typical CIDP represents a milder form of complete typical CIDP; however, its other disease characteristics, including treatment response, are similar, highlighting the importance of its proper prompt recognition as CIDP. Impairments beyond motor weakness, such as more diffuse proprioceptive loss, might play a role in the disability of patients with incomplete typical forms of CIDP.

## 1 Introduction

Chronic inflammatory demyelinating polyneuropathy (CIDP) presents, in its commonest subtype, known as the “typical” form, with symmetric proximal and distal weakness of the four limbs and large fiber sensory loss [1]. The typical nature of CIDP is established mainly by the pattern of weakness, with mandatory proximal involvement, rather than by the sensory deficits which may be variable in their affected modalities and severity. Variant forms of CIDP include focal or multifocal, pure/predominant motor, or sensory forms as well as distal presentations [1].

Recently, unclassified forms of CIDP have been described from the large national Italian database [2]. These included what were reported as “incomplete typical” forms, with (i) distal arm sparing, (ii) proximal leg sparing, and (iii) proximal arm sparing. Other unclassified presentations included paraparetic forms, arbitrarily separated from those labeled as “incomplete typical,” as well as distal leg forms with cranial involvement (described as “cranial‐predominant CIDP”).

CIDP poses a diagnostic challenge; misdiagnosis and diagnostic delay are well reported in different populations [3, 4, 5]. As the diagnosis is primarily clinical, unclassified presentations may add to these difficulties. Furthermore, different CIDP variants involve varying degrees of disability and also possibly differing treatment responses [6]. Attempting to ascertain the characteristics of unclassified subtypes may hence be useful for management.

We aimed to verify the occurrence and determine the frequency of incomplete typical forms of CIDP from a cohort of de novo, treatment‐naïve consecutive subjects attending our service. We determined the clinical, electrophysiological, and therapeutic features of this sub‐group in comparison with those presenting with the complete typical CIDP phenotype.

## 2 Methods

We retrospectively reviewed electronic records of all consecutive patients presenting with a clinical diagnosis of “CIDP” meeting EAN/PNS 2021 Guidelines [1], seen at the Inflammatory Neuropathy Service, University Hospitals Birmingham, United Kingdom. The study period was between July 2014 and January 2024. We selected exclusively de novo, treatment‐naive subjects, with a symmetrical form of CIDP, involving proximal muscle groups of upper and/or lower limbs and sensory changes in two or more limbs [1], at first assessment at our center.

We determined (i) demographics, (ii) weakness distribution, with Medical Research Council sum scores (MRCSS) for eight paired muscle groups (shoulder abductors, elbow flexors, wrist extensors, finger abductors, hip flexors, knee flexors, ankle dorsiflexors, and great toe extensors) at presentation, (iii) pre‐treatment Overall Neuropathy Limitation Score (ONLS) [7], (iv) disease duration from onset to time of treatment initiation at our center, and (v) acuteness of presentation. For the purposes of this study, we arbitrarily considered that all symmetrical forms of CIDP which involved proximal muscle groups of upper and/or lower limbs represented a form of typical CIDP. We categorized, using previously used definitions, with some modifications, incomplete typical forms as follows: (a) sparing of distal upper limb muscles, (b) sparing of proximal lower limb muscles, (c) sparing of proximal upper limb muscles, (d) paraparetic form with sparing of proximal and distal upper limb muscles and weakness of proximal and distal lower limb muscles, (e) pure proximal form, involving only proximal muscles of upper and lower limbs but sparing distal muscles.

We determined phenotype post‐treatment, categorizing the included subjects as having (i) complete normalized motor function (MRCSS 80), (ii) switched to an alternative phenotype, or (iii) maintained the same phenotype.

We also aimed to determine, secondarily, associations of pre‐treatment electrophysiological measures with response. We recorded pre‐treatment (i) summated compound muscle action potential (CMAP) amplitudes, adding the distal CMAP amplitudes evoked for unilateral median/ulnar/common peroneal/tibial nerves, (ii) summated sensory nerve action potential (SNAP) amplitudes, adding unilateral sural and radial SNAP amplitudes [8, 9, 10]. We also recorded for the aforementioned motor nerves the conduction velocity, distal latency, distal CMAP duration, conduction block at the forearm/foreleg, temporal dispersion at the forearm/foreleg, and minimum F‐wave latency.

We ascertained the maximum treatment effect attained (best total ONLS), and maintained for at least 3 months for all subjects, as well as the time taken to reach it.

Statistical analyses were performed with R version 4.3.1. Comparison of proportions was performed by Chi‐square tests, and comparison of means was performed by *T*‐tests. Correlations were performed by Pearson's correlation tests. Statistical significance was set at two‐tailed *p* < 0.05. Change in phenotype over time was illustrated using a Sankey diagram and a survival plot.

## 3 Results

From a cohort of 214 consecutive subjects registered at our service with a diagnosis of “suspected CIDP,” we identified 64 subjects meeting the inclusion criteria for the current study. Characteristics of subjects with typical and incomplete typical forms are detailed in Table 1. Demographics, mode of onset, disease duration at diagnosis and last follow‐up, comorbidities, treatment response, and electrophysiological characteristics were mostly similar between the two groups. However, motor weakness and disability pre‐treatment were significantly milder in the incomplete typical CIDP compared to those with complete typical CIDP.

**TABLE 1:** Comparison of clinical characteristics between subjects with complete vs. incomplete typical CIDP.

Characteristics	Whole (*n* = 64)	Complete typical CIDP (*n* = 45)	Incomplete typical CIDP (*n* = 19)	*p*
Age (years), mean (SD)	60 (15)	61 (16)	56 (11)	0.15
Sex (female), *n* (%)	24 (38%)	19 (42%)	5 (26%)	0.36
Phenotype at diagnosis, *n* (%)				< 0.001***
Complete typical	45 (70%)	45 (100%)		
Proximal arm sparing	12 (19%)		12 (63%)	
Distal arm sparing	3 (4.7%)		3 (16%)	
Paraparetic	3 (4.7%)		3 (15.8%)	
Pure proximal	1 (1.6%)		1 (5.3%)	
Acute onset, *n* (%)	15 (23%)	11 (24%)	4 (21%)	> 0.99
Disease duration at diagnosis (months), mean (SD)	22 (36)	24 (40)	16 (23)	0.29
Follow‐up duration (months), mean (SD)	71 (44)	70 (40)	71 (46)	0.98
Comorbidity, *n* (%)	8 (13%)	5 (11%)	3 (16%)	0.92
Diabetes, *n* (%)	17 (27%)	11 (24%)	6 (32%)	0.78
Pre‐treatment ONLS, mean (SD)	6 (3)	6 (3)	4 (2)	0.002**
Pre‐treatment MRCSS, mean (SD)	58 (12)	55 (13)	63 (8)	0.008**
Time to maximal improvement (months), mean (SD)	12 (10)	11 (10)	12 (10)	0.81
Response rate, *n* (%)				
IVIg	38 (69%)	25 (64%)	13 (81%)	0.35
Corticosteroids	12 (48%)	10 (56%)	2 (29%)	0.44
Plasma exchange	13 (81%)	11 (92%)	2 (50%)	0.27
Treatment withdrawal, *n* (%)	31 (48%)	25 (56%)	6 (32%)	0.14
Summated CMAP (mV), mean (SD)	16 (11)	15 (11)	18 (10)	0.29
Summated SNAP (μV), mean (SD)	10 (11)	10 (11)	11 (12)	0.67

** *p* < 0.01.

*** *p* < 0.001.

**FIGURE 1:**
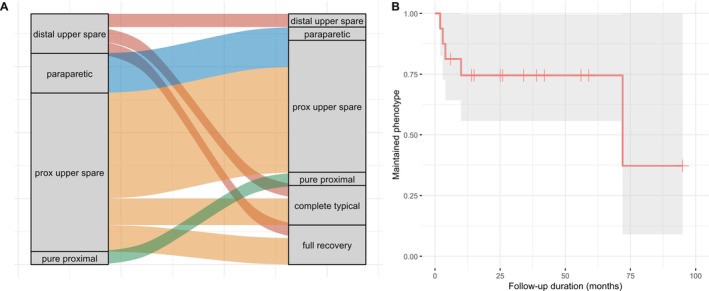
Phenotype switch in incomplete typical CIDP patients. (A) Sankey diagram showing the phenotype pre‐ and post‐treatment. (B) Kaplan–Meier curve illustrating phenotype changes over time. A drop in the curve occurs when the phenotype evolves into either the complete typical form or the incomplete typical form affecting a broader range of limbs. Censored data indicate the duration of follow‐up in patients who maintained their initial phenotype throughout the follow‐up.

The patterns of phenotype changes over time in 19 subjects with incomplete typical CIDP are illustrated in Figure 1. Among them, three evolved into the complete form over time, and three fully recovered in terms of disability (ONLS = 0) and strength (MRCSS = 80). Eleven remained in their original phenotype, while two patients with the paraparetic subtype developed distal upper limb involvement, transitioning into a proximal upper limb‐sparing form. Mean time for evolution from the initial phenotype to any other was 18.2 months (range 2–72). There was no association between pre‐treatment disease duration and evolution into a more widespread form of typical CIDP (*p* = 0.41).

In terms of electrophysiological characteristics, summated CMAPs and summated SNAP sum scores were similar between both groups. No significant differences were observed in any of the other electrophysiological motor or sensory parameters evaluated.

In the complete typical CIDP group, pre‐treatment MRCSS and ONLS scores were highly correlated (*r*: −0.75, *p* < 0.001), as were post‐treatment MRCSS and ONLS (*r*: −0.75, *p* < 0.001). However, in the incomplete typical CIDP group, only post‐treatment scores were correlated (*r*: −0.79, *p* < 0.001), whereas pre‐treatment scores were not (*r*: −0.12, *p* = 0.62).

## 4 Discussion

In our cohort, we found that nearly a third of subjects with newly diagnosed typical CIDP, defined as those with symmetric proximal weakness in the upper limbs and/or lower limbs, had an incomplete presentation. Based on detailed motor examinations, the majority of these incomplete typical presentations exhibited proximal upper limb sparing, which frequently progressed over time, eventually switching to the complete typical phenotype.

Consistent with previous reports, pre‐treatment disability and motor strength were significantly milder in the incomplete form. Prior studies have reported a higher response rate to IVIg in the incomplete form (81%) compared to the complete form (65%). While our data showed a very similar trend (81% vs. 64%), the difference was not statistically significant for all three types of first‐line treatments, likely due to the small sample size. A recent study reported on single‐limb variants of CIDP at onset [11]. These were described in 11% of variant cases, with subsequent extension of the disease to more nerves in the majority (13/16; 81.3%). Interestingly, earlier treatment had been initiated in the cases that remained monotruncular, suggestive of a possible effect on disease progression. However, a similar protective effect of early treatment could not be demonstrated in our subjects with incomplete typical CIDP.

Of note, we found no correlation between pre‐treatment MRCSS and ONLS in incomplete typical CIDP, as opposed to complete typical CIDP. These findings suggest that, compared to typical CIDP, other modalities, such as proprioception, may contribute significantly to disability in incomplete typical CIDP. Detailed clinical and functional evaluations of larger cohorts, including the extent and severity of proprioceptive loss, may in future help better understand the respective contributions of motor and sensory dysfunctions in the disability of this sub‐group.

Our study is limited by the small number of patients and the retrospective single‐center design, which may be associated with the absence of proximal leg sparing or cranial nerve predominant forms in our cohort. Also, outcome measures validated in CIDP, such as inflammatory Rasch‐built overall disability scale or grip strength, or those reflecting proprioceptive deficit or ataxia were not considered. The observation that subjects with incomplete typical CIDP exhibited comparable treatment response rates to those of the complete form underscores the importance of prompt recognition and treatment of this subgroup.

## Author Contributions

**Young Gi Min:** writing – original draft, writing – review and editing, formal analysis, visualization, methodology. **Irad Ahmed:** data curation, investigation, supervision, writing – review and editing. **Christina Englezou:** writing – review and editing. **Yusuf A. Rajabally:** writing – review and editing, writing – original draft, conceptualization, methodology, data curation, validation, investigation, project administration.

## Ethics Statement

This study was part of a retrospective Clinical Audit approved and registered by the University Hospital Birmingham NHS Foundation Trust Clinical Audit Service (CARMS no. 20702, October 23, 2023). Clinical Audit does not require Ethics Committee approval in the United Kingdom. We confirm that we have read the Journal's position on issues involved in ethical publication and affirm that this report is consistent with those guidelines.

## Conflicts of Interest

Y.A.R. has received consultancy honoraria from Sanofi, Janssen, Argenx, LFB, Polyneuron, Grifols, Takeda, and Dianthus, has received educational sponsorships from LFB and CSL Behring, and has obtained research grants from LFB. The other authors declare no conflicts of interest.

## Data Availability

The data that support the findings of this study are available from the corresponding author upon reasonable request.
